# Defining the reducing system of the NO dioxygenase cytoglobin in vascular smooth muscle cells and its critical role in regulating cellular NO decay

**DOI:** 10.1074/jbc.RA120.016394

**Published:** 2020-12-20

**Authors:** Govindasamy Ilangovan, Sahar A. Khaleel, Tapan Kundu, Craig Hemann, Mohamed A. El-Mahdy, Jay L. Zweier

**Affiliations:** 1 Department of Internal Medicine, Division of Cardiovascular Medicine, and the EPR Center, Davis Heart & Lung Research Institute, College of Medicine, The Ohio State University, Columbus, Ohio, USA; 2 Department of Pharmacology and Toxicology, College of Pharmacy, Al-Azhar University, Cairo, Egypt

**Keywords:** cell signaling, cytoglobin, nitric oxide, nitric oxide dioxygenation, nitric oxide metabolism, redox regulation, vascular relaxation, vascular smooth muscle cells, Asc, Ascorbate, B5, Cytochrome b5, B5R, Cytochrome b5 reductase, CPR, Cytochrome p450 reductase, Cygb, Cytoglobin, eNOS, Endothelial nitric oxide synthase, FBS, Fetal bovine serum, Hb, Hemoglobin, Mb, Myoglobin, NADH, Nicotinamide adenine dinucleotide (reduced), NO, Nitric oxide, NOD, Nitric oxide dioxygenation, sGC, Soluble guanine cyclase, SMCs, Smooth muscle cells, SOD, Superoxide dismutase, V_NO_, Rate of NO dioxygenation

## Abstract

In smooth muscle, cytoglobin (Cygb) functions as a potent nitric oxide (NO) dioxygenase and regulates NO metabolism and vascular tone. Major questions remain regarding which cellular reducing systems regulate Cygb-mediated NO metabolism. To better define the Cygb-mediated NO dioxygenation process in vascular smooth muscle cells (SMCs), and the requisite reducing systems that regulate cellular NO decay, we assessed the intracellular concentrations of Cygb and its putative reducing systems and examined their roles in the process of NO decay. Cygb and the reducing systems, cytochrome b5 (B5)/cytochrome b5 reductase (B5R) and cytochrome P450 reductase (CPR) were measured in aortic SMCs. Intracellular Cygb concentration was estimated as 3.5 μM, while B5R, B5, and CPR were 0.88, 0.38, and 0.15 μM, respectively. NO decay in SMCs was measured following bolus addition of NO to air-equilibrated cells. siRNA-mediated knockdown experiments indicated that ∼78% of NO metabolism in SMCs is Cygb-dependent. Of this, ∼87% was B5R- and B5-dependent. CPR knockdown resulted in a small decrease in the NO dioxygenation rate (V_NO_), while depletion of ascorbate had no effect. Kinetic analysis of V_NO_ for the B5/B5R/Cygb system with variation of B5 or B5R concentrations from their SMC levels showed that V_NO_ exhibits apparent Michaelis–Menten behavior for B5 and B5R. In contrast, linear variation was seen with change in Cygb concentration. Overall, B5/B5R was demonstrated to be the major reducing system supporting Cygb-mediated NO metabolism in SMCs with changes in cellular B5/B5R levels modulating the process of NO decay.

Nitric oxide (NO), generated through activation of endothelial NO synthase (eNOS) in blood vessels, causes smooth muscle relaxation with vasodilation that enhances tissue perfusion (1, 2, 3). The NO, formed in the endothelium, diffuses to reach the vascular smooth muscle layer of blood vessels. In smooth muscle cells (SMCs), soluble guanylate cyclase (sGC) binds NO, with the formation of cGMP, which induces SMC relaxation and vasodilation (1, 2). While the process of NO formation and signaling has been extensively studied and is well understood, many questions remain regarding the process of NO decay and how this is regulated (4, 5, 6, 7, 8). Although NO is critical to mediate vessel relaxation, if its levels were to rise too high, vessel tone would greatly decrease resulting in physiological hypotension (9). In excess, NO can be directly deleterious or exert toxicity through the formation of reactive nitrogen species such as the potent oxidant peroxynitrite (10, 11, 12, 13).

Globins contain redox active heme centers with either 5- or 6-coordinate iron and, in the presence of O_2_, degrade NO by NO dioxygenation (NOD) with the resulting formation of NO_3_^−^ (5, 7, 14, 15, 16, 17). The efficiency of NOD activity of various mammalian globins, hemoglobin (Hb), myoglobin (Mb), cytoglobin (Cygb), and neuroglobin (Ngb) varies based on differences in their heme center and overall structure. Their role in NOD in different cell types depends on their expression levels, as well as the availability of suitable reducing systems to support O_2_-dependent NOD (5, 7, 8, 18, 19, 20, 21, 22). Endothelium-generated NO that diffuses into the lumen of vessels is metabolized by the hemoglobin-rich erythrocytes, which contain high millimolar levels of oxy-Hb (23). However, questions have remained regarding the metabolism of NO as it diffuses into the smooth muscle containing vessel wall. Until recently, the underlying mechanism of NO metabolism in smooth muscle was unclear, with myoglobin and cytoglobin proposed as vascular NOD, and Hb-α was recently proposed to have a role in NOD in myoendothelial junctions (8, 24, 25, 26, 27).

Recent studies have reported that Cygb is the major NOD in vascular SMCs and in the vessel wall (5). This was found to be due to the much higher expression levels of Cygb than other globins such as myoglobin or Hb that were only present in trace levels 40- to >200-fold lower than Cygb, respectively (5, 7). In addition, from studies of isolated protein systems, the rate of NOD by Cygb has been shown to be much higher than these other globins, due to its more rapid reduction rate with a variety of reductants, such as ascorbate, or reducing systems, such as P450 reductase (CPR) or cytochrome b5/b5 reductase (B5/B5R). Cygb was also shown to be unique in the O_2_-sensitivity of its NOD function, whereby it can provide O_2_ sensing with a lower NOD rate under conditions of physiological hypoxia (7, 16, 28). Further studies in Cygb knockout mice demonstrated that Cygb has a key role in the regulation of vascular tone and NO decay. In these mice with genetic deletion of Cygb, vascular dilation was seen along with decreased systemic blood pressure and lower systemic vascular resistance (5).

The NOD function of Cygb and other globins depends on the capacity to generate and regenerate the catalytic oxy-ferrous species (Fe^2+^O_2_) that mediates the dioxygenation of NO. The Fe^3+^ heme of a given globin is reduced to Fe^2+^ heme by chemical or enzymatic reducing systems such as ascorbate (Asc) (4, 7, 16, 28, 29, 30), CPR (5), and B5/B5R (5, 16, 29, 31). Once the heme is reduced, O_2_ rapidly binds to the Fe^2+^ to form the oxy-ferrous species, Fe^2+^O_2_. Fe^2+^O_2_ reacts with NO to generate NO_3_^−^, with regeneration of Fe^3+^ heme, and this catalytic cycle continues (7, 8). Therefore, the reduction rate of a given globin determines its efficiency and overall NOD activity (7, 28, 29). From prior studies of Cygb, both ascorbate and the enzyme reducing systems B5/B5R and CPR are highly effective in reduction of the heme; however, much higher levels of ascorbate are required than for the enzyme systems to support a given rate of reduction. Since ascorbate can be present up to millimolar levels in cells, while the enzyme reducing systems were thought to be well below micromolar, it had been hypothesized that ascorbate as well as the enzyme reducing systems could play a role in supporting the process of NOD in SMCs (7, 16). More recently, the role of ascorbate in Cygb reduction has been questioned (29). In order to understand the process of NOD in SMCs and how this may regulate vascular function, it is important to know the concentration of Cygb and each reducing system that supports this process. With this information, one can then model and predict the rate of NO decay in these cells and how this may modulate vascular function and tone.

In spite of detailed *in vitro* studies measuring the NOD activity of Cygb, to date there is a lack of knowledge regarding the specific reducing systems that regulate its NOD activity in SMCs and their relative importance. These reducing systems are of critical importance for regeneration of active oxy-ferrous Cygb required for NO dioxygenation. There is a critical lack of knowledge on the expression levels of Cygb and its requisite reducing systems in SMCs and how this regulates NOD activity. This information on the redox regulation of Cygb in SMCs is required to understand its process of NO dioxygenation and how this regulates NO-induced vasorelaxation with regulation of vascular tone.

Therefore, in the current work, studies were performed in mouse aortic SMCs to measure and quantitate the levels of Cygb as well as the major enzyme reducing systems including B5/B5R and CPR that are proposed to regulate its NOD function. The effects of knockdown of each of these proteins involved in NO metabolism were determined and also compared with the effects of depletion or supplementation of ascorbate. Our studies establish that Cygb provides most of the NOD function of SMCs, and B5/B5R was shown to be its major reducing system, while CPR has a significant but lesser role. In contrast, ascorbate depletion had no significant effect on NO metabolism. Based on the measured levels of Cygb and its reducing system in SMCs, we experimentally model the effects of changes in these levels on the rate of NO decay and consider the effects this will have on the process of NO metabolism that regulates vascular tone.

## Results

### NO dioxygenation in aortic SMCs

**Figure 1:**
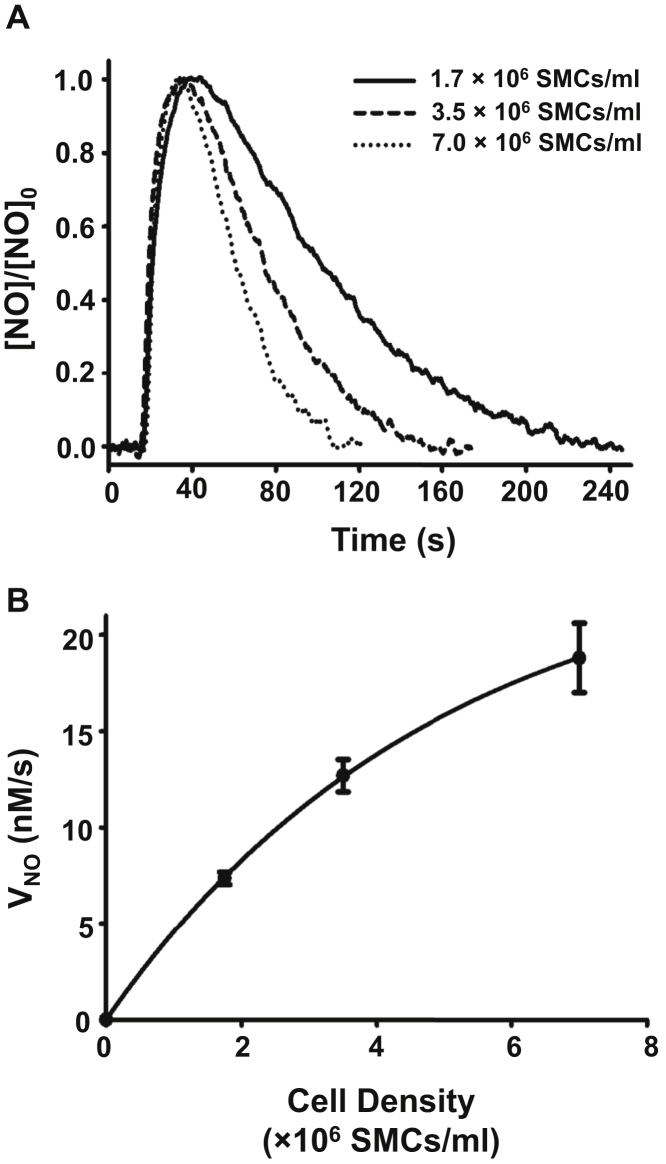
**Effect of SMC number on NO decay.***A*, NO consumption by mouse aortic SMCs at three cellular densities (1.7, 3.5, and 7 ×10^6^ SMCs•ml^−1^). NO was injected into the solution to achieve an initial concentration of 1.0 μM. *B*, graph of the rate of NO decay (*V*_NO_) by 1.75, 3.5, and 7 × 10^6^ SMCs•ml^−1^. Values shown are the means ± SD with n = 4.

It is expected that the rate of NO metabolism in cell suspensions would be proportional to the density of the cells and NO metabolizing proteins, with the greater the cell number, the higher the rate of NOD and decay. However, there may be an upper limit on the cell density to prevent cell aggregation as well as metabolic ischemia. In order to determine the best range of cell density values in suspension to provide reproducible measurements of NO decay rates, initial experiments were performed to measure the rate of NO metabolism as a function of the number of SMCs per volume (Fig. 1). We evaluated three different cell densities, 1.75, 3.5, and 7 × 10^6^ cells•ml^−1^. NO decay was measured following addition of a bolus of NO sufficient to reach a 1 μM initial NO concentration in the 2 ml cell suspension. As we have previously shown, using quantitative HPLC, more than 90% NO metabolism occurs in SMCs through the NOD reaction with the production of nitrate (NO_3_^−^) (5). Typical tracings of the NO concentration change over time are shown for the three different cell concentrations (Fig. 1*A*). With higher numbers of SMCs/ml, more rapid decay of NO was seen. Quantitative measurement of rates of NO metabolism (*V*_NO_) was performed, from the slope of the initial decay (V_NO_=Δc/Δt), as previously described (7). The data from repeat NO decay measurements corrected for the rates of NO decomposition (V_d_) in cell-free buffer was used to generate plots of *V*_NO_ *versus* cell density (Fig. 1*B*). With 1.75 and 3.5 × 10^6^ cells•ml^−1^ a clear increase in *V*_NO_ was seen with cell number. With a further doubling of cell numbers to 7 × 10^6^ cells•ml^−1^, while a further increase was seen, the *V*_NO_ per cell decreased as the curve started to plateau (Fig. 1*B*). This may be due to concentration-dependent cell aggregation and settling, or the limited availability of metabolic substrates for this high cell density in the measurement chamber. Based on this data, we utilized a cell density of 3.5 × 10^6^ cells•ml^−1^ in our subsequent studies to maintain a high rate of NO decay while avoiding the saturation seen with higher cell densities.

**Figure 2:**
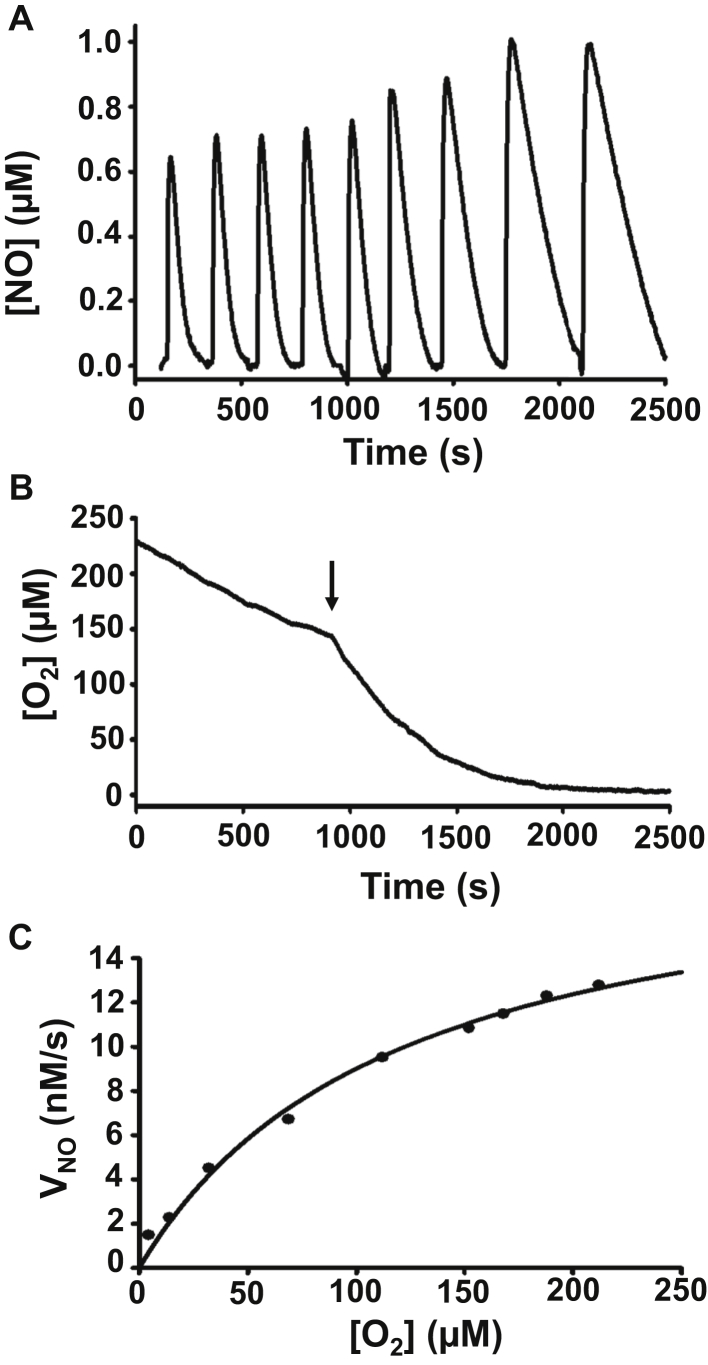
**Effect of O**_**2**_**level on the rate of NO metabolism by SMCs.***A*, measurements of rates of NO consumption by mouse aortic SMCs at varying O_2_ concentrations. NO (1.0 μM) was repeatedly added to the solution containing 3.5 × 10^6^ SMCs•ml^−1^. *B*, the solution was initially aerated with flow of purified air over the solution surface and the gas flow switched to argon at the time designated by the arrow (↓) to accelerate the decline in the dissolved O_2_ concentration in the solution. *C*, plot of the rate of NO consumption by SMCs at varying O_2_ levels. The data points were obtained by repeated addition of 1.0 μM NO to the solution containing 3.5 × 10^6^ SMCs•ml^−1^.

While O_2_ is required for NOD with O_2_ availability critical for NO metabolism by Cygb, the dependence of NO decay on the O_2_ level in SMCs has not been previously measured. We have previously observed in isolated protein studies using purified Cygb that the NOD reaction is O_2_-dependent with apparent Michaelis–Menten kinetics (7). Therefore, studies were performed to measure the effect of O_2_ levels on NO metabolism by SMCs. For these studies, NO and O_2_ were simultaneously measured by polarographic electrodes (Fig. 2, *A*–*B*). In these experiments, repeated bolus injections of 1 μM NO were added to 3.5 × 10^6^ cells•ml^−1^ at ∼ 4 min intervals with simultaneous measurements of the NO and O_2_ concentrations present in the cell suspensions. After several recordings of the cell suspensions with flow of purified air over the solution surface, the air stream was stopped and then flow of argon initiated to accelerate the decline in O_2_ to near anaerobic levels. NO metabolism rate following each bolus addition of NO was determined and plotted against the corresponding O_2_ concentration as determined from the simultaneous measurements, as shown in Figure 2*C*. The plot of NO consumption with respect to [O_2_] shows a hyperbolic dependence with progressive decrease as the O_2_ concentration drops below 200 μM. Thus, with physiological hypoxia, as O_2_ levels decline, the NOD rate would also decline with lower rate of NO metabolism that could trigger a compensatory vasodilation.

### Cygb expression and NOD activity in SMCs

**Figure 3:**
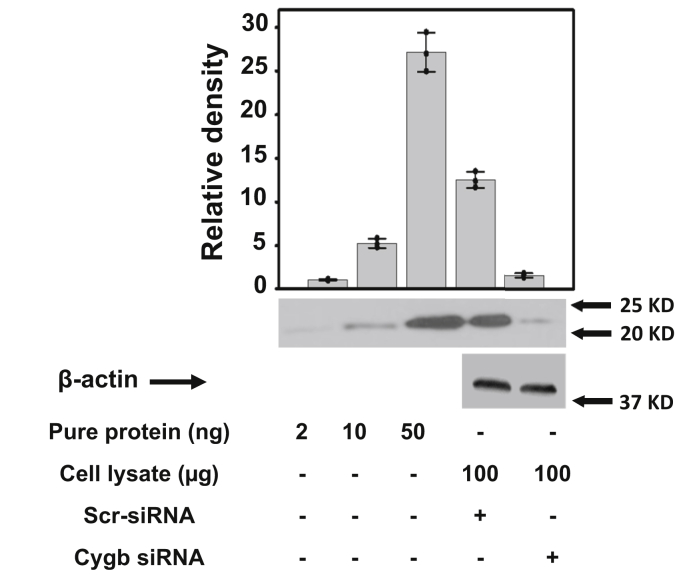
**Expression level of Cygb in SMCs.** Western blots of Cygb obtained from scr-RNA and Cygb siRNA-treated mouse aortic SMCs. The first three bands correspond to known amounts of purified Cygb. Bands 4 and 5 are from scr-RNA and Cygb siRNA-treated cells, respectively. The bar graph shows the mean value ±SD for n = 3 to 5. From the ratio of measured Cygb to total cellular protein concentration, the cellular concentration of Cygb was estimated to be 3.5 μM.

The level of Cygb expression in SMCs and the effectiveness of siRNA treatment in knocking down its expression level were determined by quantitative immunoblotting, using known concentrations of purified Cygb as standard. Representative blots and quantitative analyses of blot densities are shown (Fig. 3). The first three bands correspond to known amounts of purified Cygb. Bands 4 and 5 were obtained from equal loading of homogenates of scr-RNA treated cells and matched Cygb siRNA-treated cells. From the band intensities of known concentrations of Cygb, blotted along with cell lysates, the intracellular concentration of Cygb in mouse SMCs was estimated to be 3.5 ± 0.2 μM. siRNA treatment of cells successfully reduced the Cygb expression by 87% in SMCs.

**Figure 4:**
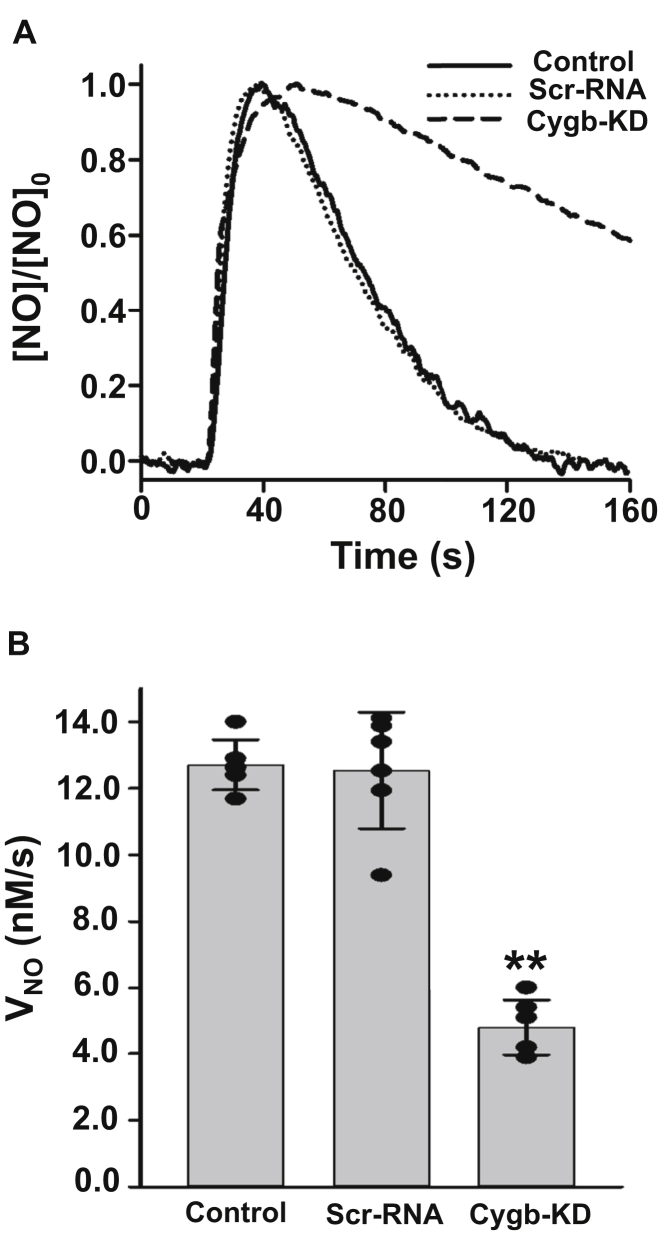
**Effect of Cygb knock down on NO metabolism in SMCs.***A*, NO consumption by control, scr-RNA, or Cygb siRNA-treated mouse aortic SMCs. NO was injected as in Figure 1. *B*, graph of the rate of NO decay (*V*_NO_) measured from control, scr-RNA, and siRNA-treated SMCs. A cell density of 3.5 × 10^6^ SMCs•ml^−1^ was maintained in all groups. Values shown are means ± SD with n = 6. ∗∗Significant from scr-RNA at *p* < 0.001.

NO metabolism both in control SMCs and in those with Cygb knockdown was measured, as described above, following bolus addition of 1 μM NO to the SMCs with monitoring of both NO concentration and O_2_ level with the chamber open to air. NO metabolism in SMCs was determined by following NO decay with 3.5 × 10^6^ cells•ml^−1^. Figure 4*A* shows representative tracings of the change in NO concentration over time in siRNA-treated cells compared with untreated control cells and the corresponding scrambled siRNA (scr-RNA)-transfected cells. Quantitative analyses of *V*_NO_, from a series of repeat measurements, are presented in Figure 4*B*. NO tracings for siRNA-treated cells showed much slower decay than in control cells or scr-RNA-treated cells. NO decay rate in control cells was 12.7 ± 0.3 nM•s^−1^. With scr-RNA treatment of SMCs, there was no significant change with a value of 12.5 ± 0.5 nM•s^−1^ (Fig. 4*B*). The *V*_NO_ in siRNA-treated Cygb knockdown aortic SMCs was decreased by 68% with a value of 4.1 ± 0.2 nM•s^−1^. With the 87% knockdown of Cygb in siRNA-treated cells (Fig. 3), this indicates that at least 78% of NO metabolism is Cygb-dependent in the mouse aortic SMCs studied. Thus, as reported previously in other vascular SMC lines (5), Cygb was confirmed to be the major globin protein mediating NO decay.

### Effect of Cygb-reducing systems on NOD in SMCs

**Figure 5:**
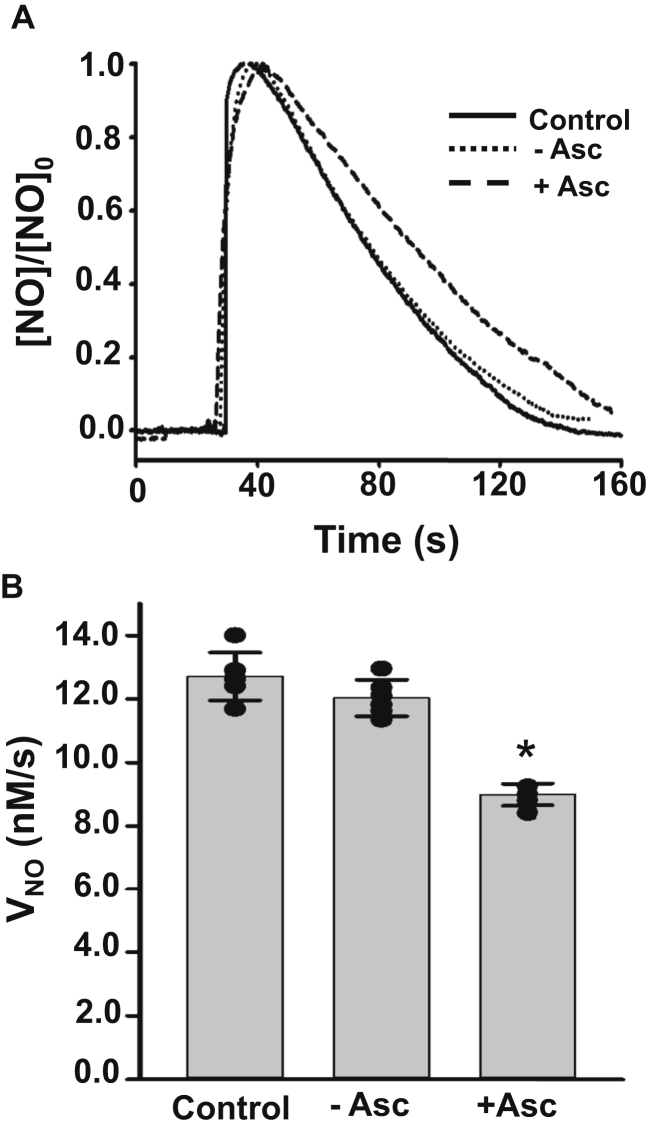
**Effect of ascorbate deprivation (-Asc) and supplementation (+Asc) on NO metabolism in SMCs.***A*, NO consumption by control and Asc-deprived mouse aortic SMCs. NO was injected as in Figure 1. *B*, graph of the rate of NO decay (*V*_NO_) by control, −Asc, and +Asc SMCs determined from the initial slopes of the NO decay curves in repeat measurements. A cell density of 3.5 × 10^6^ SMCs•ml^−1^ was used in all the groups. Values shown are means ± SD (n = 6), ∗*p* < 0.01.

Next, we evaluated the role of different cellular reducing systems that have been previously shown to reduce Cygb in isolated protein studies and have been proposed to be involved in its reduction in SMCs. As various reducing systems, including ascorbate (7, 29), CPR (7) and B5/B5R (29, 31), have been reported to be effective reducing systems to reduce Cygb-Fe^3+^ to generate catalytically active Cygb-Fe^2+^-O_2_, we determined the effect of depletion of each of these on NOD activity of Cygb in SMCs. To assess the effect of ascorbate concentrations on intracellular NO metabolism, we compared the effect of depleting or supplementing ascorbate levels in these cells. SMCs were studied that were cultured in standard medium supplemented with 10% FBS with an intracellular ascorbate concentration of ∼50 μM (32). For depletion of intracellular ascorbate, we maintained the SMC cultures in ascorbate-free media for 48 h prior to cell harvesting. In an effort to increase cellular ascorbate levels, cells were maintained in media supplemented with 5 mM ascorbate for 48 h, which would be expected to greatly increase cellular ascorbate levels (33). Figure 5*A* shows tracings of the NO concentration over time for control, ascorbate-deprived, and ascorbate-supplemented cells. No significant difference was seen with the ascorbate-deprived cells. *V*_NO_ was determined from a series of repeat experiments in control and ascorbate-deprived cells, as shown in Figure 5*B*. Intracellular ascorbate deprivation did not significantly change the NOD rate compared with control (11.7 ± 0.7 nM•s^−1^ and 12.5 ± 0.5 nM•s^−1^, respectively). Interestingly, in ascorbate-supplemented cells, no increase in *V*_NO_ was seen, rather a small but significant decrease occurred (9.2 ± 0.4 nM•s^−1^, ∼26% decrease compared with the control cells).

**Figure 6:**
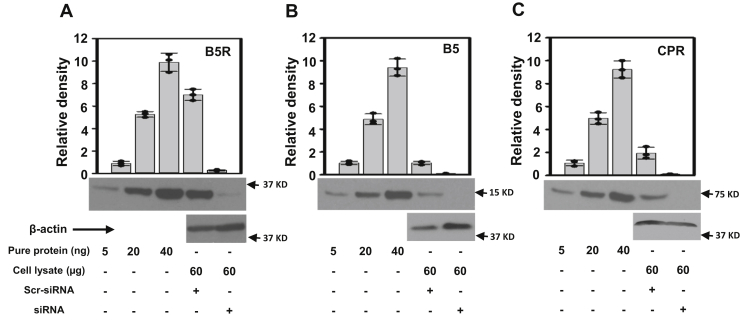
**Expression levels of B5R, B5, or CPR in SMCs**. Western blots of B5R (*A*), B5 (*B*), and CPR (*C*) obtained from scr-RNA and B5R, B5 and CPR siRNA-treated mouse aortic SMCs. The first three bands correspond to known amounts of purified B5R (*A*), B5 (*B*), or CPR (*C*). Bands 4 and 5 are from scr-RNA and B5R (*A*), B5 (*B*), or CPR (*C*) siRNA-treated cell homogenates, respectively. The bar graphs show the mean value ± SD for n = 3. From the ratio of each measured protein to total cellular protein concentration, the cellular concentrations of B5R, B5, and CPR are estimated at 0.88 μM, 0.38 μM, and 0.15 μM, respectively.

In order to decrease the concentrations of the enzyme reducing systems, B5R, B5, and CPR, we utilized their respective siRNAs. For these experiments, depletion of each of these proteins was achieved by transfection of the SMCs with the desired siRNA, and then at 48 h posttransfection immunoblotting was performed to assess the efficiency of protein knockdown. Quantitative immunoblotting was performed for each knockdown, comparing scr-RNA controls with siRNA-treated lysates, and these were blotted along with known quantities of the pure proteins (Fig. 6). From this quantitative immunoblotting, the cellular concentrations of these proteins were measured as: 0.88 ± 0.04 μM B5R; 0.38 ± 0.03 μM B5; 0.15 ± 0.02 μM CPR. B5R, B5, and CPR siRNA decreased the level of the respective protein by ∼ 90% to 95% compared with scr-RNA treatment. The measured concentrations of B5R and B5 in SMCs are seen to be higher than previously assumed (7, 16). Also, interestingly, the level of B5R measured is higher than B5. In contrast to most previously published studies with isolated protein-based assays where B5/B5R ratios are maintained at least 10:1 to maximize the electron flux for a given amount of B5R (5, 29, 31), we observe a fractional value of ∼0.43 in SMCs. Thus, interestingly, B5 levels would appear to be more limiting than B5R, and the relatively low B5/B5R ratio in SMCs might render B5 levels of importance in regulating Cygb reduction.

**Figure 7:**
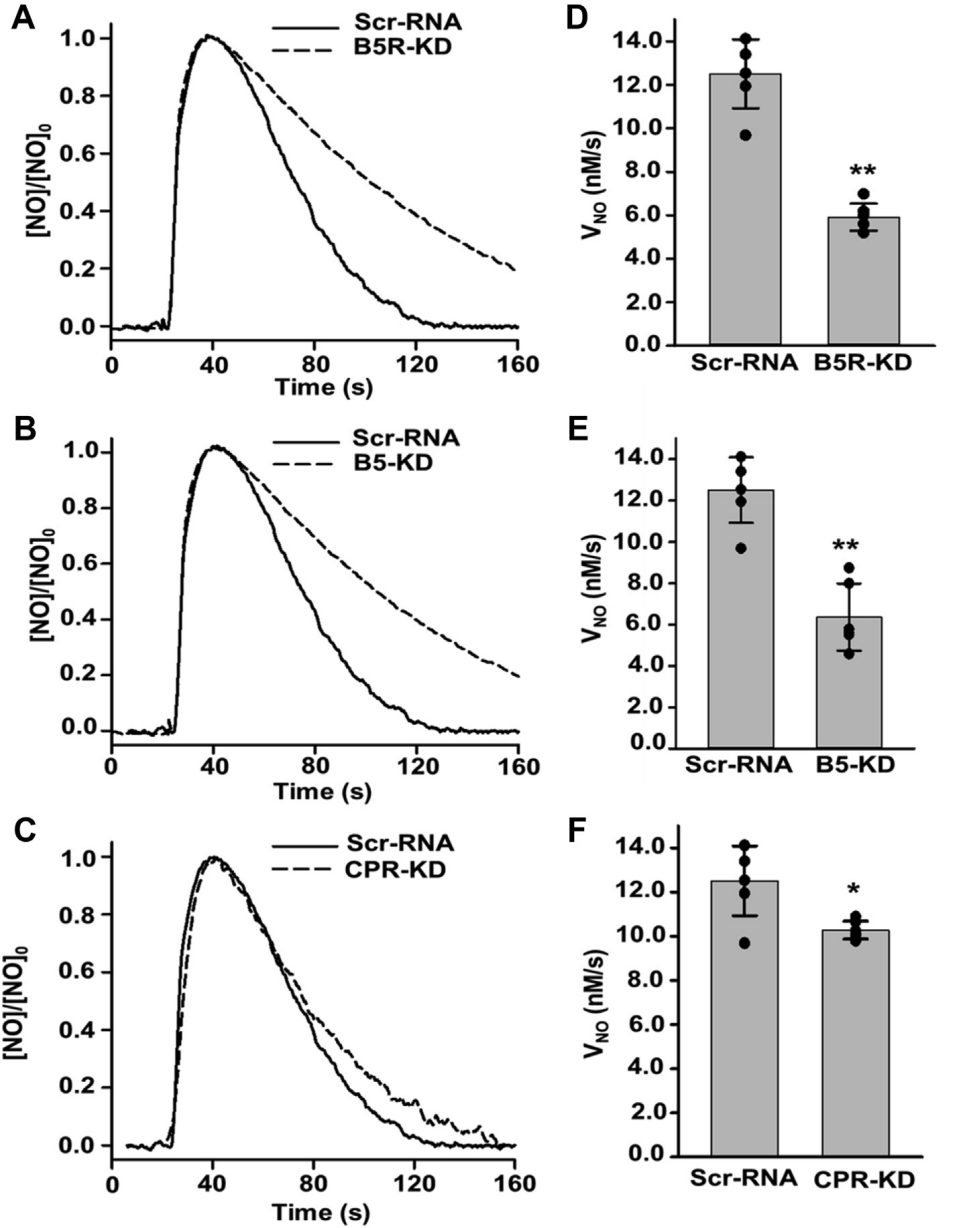
**Effect of B5R, B5, and CPR on the rates of NO consumption by SMCs.***A*–*C*, NO consumption by scr-RNA *versus* B5R (*A*), B5 (*B*), and CPR (*C*) siRNA-treated (KD) mouse aortic SMCs. A cell density of 3.5 × 10^6^ SMCs•ml^−1^ was used in all groups. NO was injected into the solution to achieve an initial concentration of 1.0 μM. Graph of the rates of NO metabolism (*V*_NO_) by scr-RNA *versus* B5R (*D*), B5 (*E*), and CPR (*F*) siRNA-treated (KD) SMCs. The bar graph shows the mean value ± SD for n = 6. ∗: Significant from scr-RNA at *p* < 0.05; ∗∗significant from scr-RNA at *p* < 0.001.

Measurements of the rate of NO consumption performed in these matched scr-RNA and siRNA-treated cells of B5R, B5, and CPR are shown in Figure 7, *A*–*C*. With knockdown of either B5R or B5, the rate of NO decay was greatly decreased compared with that in untreated control or scr-RNA-treated cells. In addition, in CPR knockdown cells, a lesser effect was seen. From a series of repeat experiments, quantitative measurements of *V*_NO_ were obtained from each group of SMCs and are summarized in Figure 7, *D*–*F*. B5R or B5 knockdown decreased the *V*_NO_ by 53% and 49%, respectively. However, in CPR siRNA-treated cells, the *V*_NO_ was decreased by only 18%. As the efficiency of knockdown was 95% and 90%, respectively, we can estimate that at least 56% or 54% of the NO consumption was B5R or B5-dependent. With the measured 95% knockdown efficiency, at least 19% of NO metabolism was CPR-dependent. Overall these results establish that B5/B5R has a major role in supporting the NOD activity of Cygb in SMCs, with a smaller contribution from CPR.

### Role of cellular B5R, B5, and Cygb levels in regulating NOD rate

**Figure 8:**
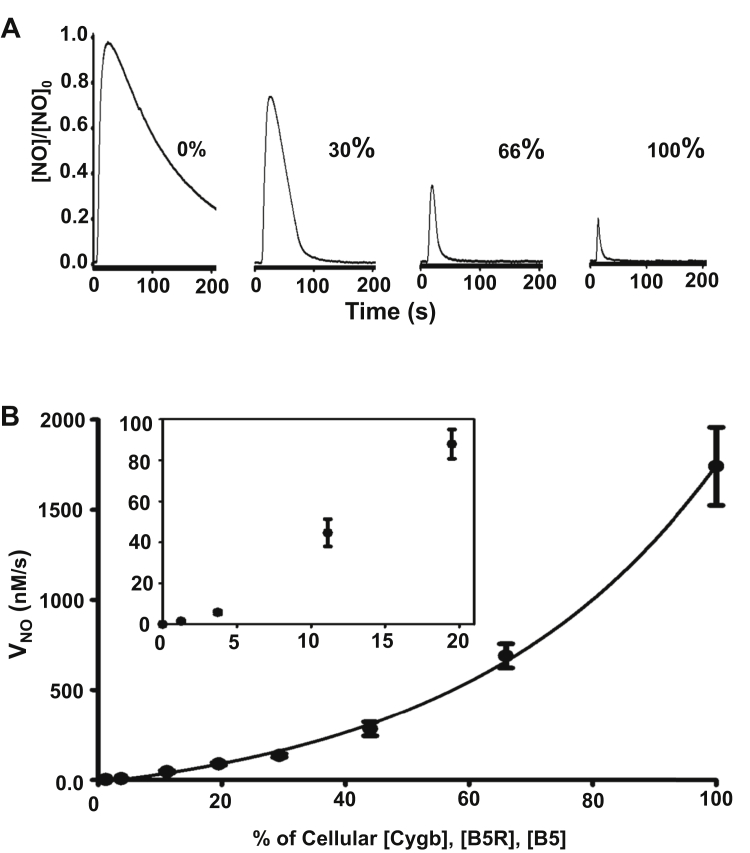
**Effect of varying cellular levels of Cygb, B5R, B5, on NO metabolism.***A*, NO consumption in the presence of 100 μM NADH and 6 μM SOD at 0%, 30%, 66%, and 100% of cellular Cygb, B5R, and B5 concentrations. *B*, graph of the NO decay rate (*V*_NO_) from repeated experiments with cellular concentrations of Cygb (3.5 μM), B5R (0.88 μM), and B5 (0.38 μM) and with subsequent serial dilutions (from 100% to 1.0%). Solid line is an exponential fit of experimental data. NO was injected with a concentration of 10 μM for 100% to 30% cellular concentrations and a concentration of 1 μM for subsequent dilutions. Final rates were normalized for 1 μM NO. Values shown are means ± SD with n = 4.

In order to assess the effects of the estimated cellular levels of B5R, B5, and Cygb on the process and magnitude of NO decay, we measured NOD activity with these purified proteins using the concentrations measured in the SMCs. First, we performed assays maintaining the same concentration and ratio of B5R (0.88 μM), B5 (0.38 μM), and Cygb (3.5 μM) as measured in the SMCs. *V*_NO_ for these concentrations of B5R, B5, and Cygb (100 μM NADH) was found to be 1741 ± 63 nM•s^−1^ with corresponding turnover of Cygb of 0.50 s^−1^ (Fig. 8). This high *V*_NO_ was roughly 150 times higher than that determined in SMC suspensions (Fig. 1). Since the packed volume of cells from suspensions with 3.5 × 10^6^ cells/ml was determined to be ∼0.7%, the predicted value for *V*_NO_ in the cell suspension would be ∼12.2 ± 0.4 nM•s^−1^, consistent with the range of values 12.0 to 12.7 nM•s^−1^ that was observed in control SMC suspensions (Figs. 1, 4 and 5). This confirms that the cellular NOD rate is comparable with that with the purified proteins, with similar *V*_NO_ at similar concentrations.

Further experiments were performed with serial dilutions of the Cygb, B5 and B5R to determine the effects of decreasing the concentration of these proteins while maintaining their relative ratios. The reaction mixture was serially diluted with buffer (from 1.5-fold dilution to ∼81-fold dilution), while NADH was maintained at 100 μM, and the NOD rates were determined (Fig. 8). While it is expected that *V*_NO_ is proportional to the Cygb concentration when the reducing system concentration is fixed (7), with serial dilutions the *V*_NO_ exponentially declined, suggesting that the high concentrations of B5/B5R measured in cells are required to maximize the rate of Cygb reduction and the resulting *V*_NO_. The *V*_NO_ values at 1.5, 2, 3, 5, 9, 27, and 81-fold dilution decreased by ∼3, 6, 13, 20, 40, 300, and 1200-fold, respectively (Fig. 8*B*). These results show that when the Cygb, B5R, and B5 concentrations are all proportionally decreased, the resulting V_NO_ exponentially declines. Thus, the high concentrations of Cygb and its B5/B5R reducing system as measured in the SMCs are required to support the high rate of NO degradation that was observed.

**Figure 9:**
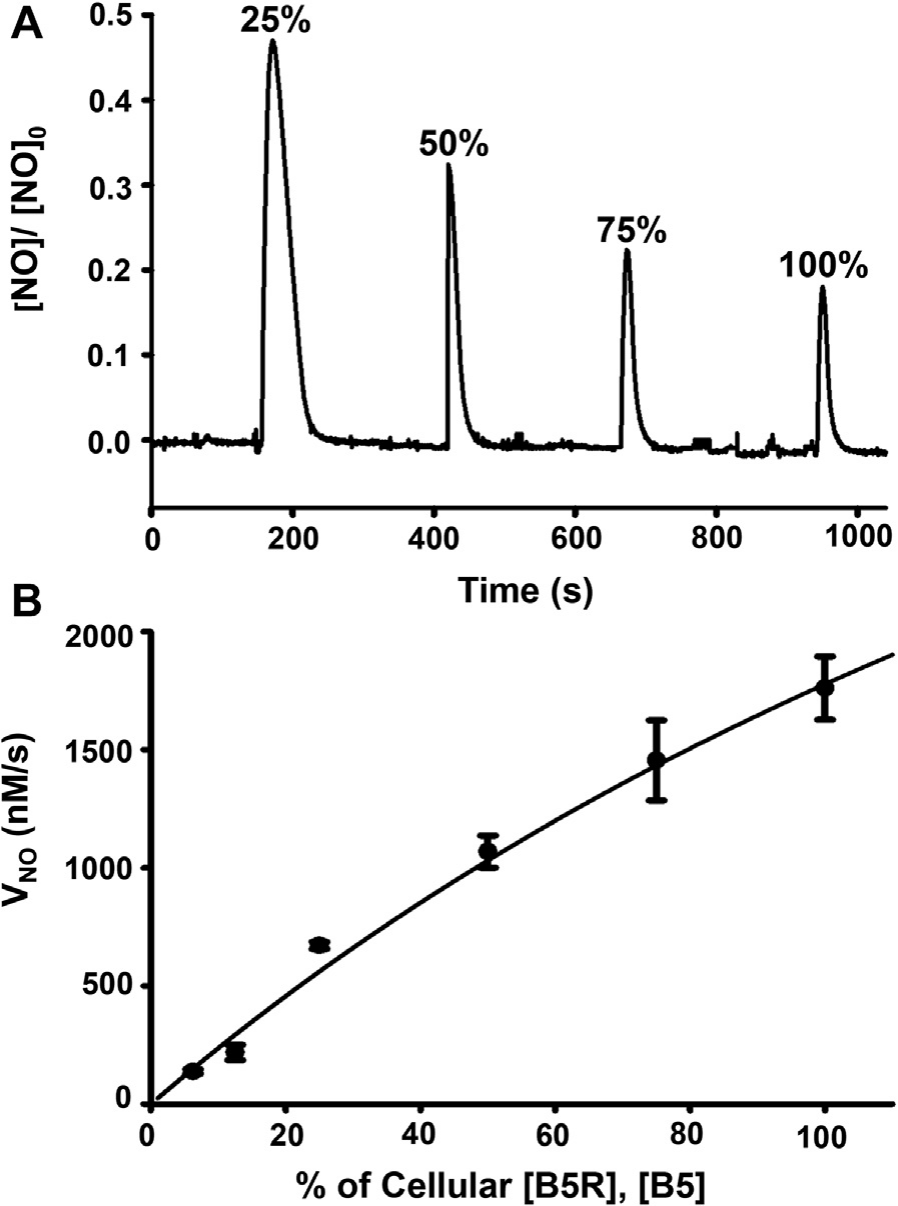
**Effect of varying B5/B5R levels on NO metabolism.** B5 and B5R concentrations were varied from the values determined in SMCs, keeping the B5/B5R ratio (0.43) constant with 3.5 μM Cygb. *A*, NO consumption from left to right of: 25; 50; 75; and 100% of cellular B5/B5R concentrations (0.88 μM and 0.38 μM, respectively). Experiments were performed and analyzed similar to Figure 8. *B*, graph of the NO decay rate (*V*_NO_) from repeated experiments in the reaction mixture (NADH 100 μM, Cygb 3.5 μM) with various B5/B5R concentrations. Values shown are means ± SD with n = 3.

Since B5/B5R was observed to be the major enzyme reducing system supporting the NO dioxygenase activity of Cygb in SMCs, additional experiments were performed to determine how decreasing the cellular levels of B5/B5R (0.88 μM B5R; 0.38 μM B5) modulates the NOD rate with fixed cellular level of Cygb (3.5 μM). In these experiments, the ratio of B5/B5R was fixed at the value of ∼2.3 as determined to be present in SMCs. The levels of B5/B5R were serially increased from 25% to 50%, 75%, and 100% of the cellular levels, while the Cygb concentration was fixed (Fig. 9*A*). With this, the observed NO peaks decreased in amplitude and width due to the increased rates of NO decay. As depicted in Figure 9*B*, with decrease in the concentrations of B5/B5R from 100% of the cellular levels to less than 10%, the rate of NO decay progressively declines. In these repeated quantitative measurements of V_NO_ for given % cellular B5/B5R levels, a hyperbolic response is seen with a trend toward saturation at higher B5/B5R levels as occur in the SMCs (Fig. 9*B*).

**Figure 10:**
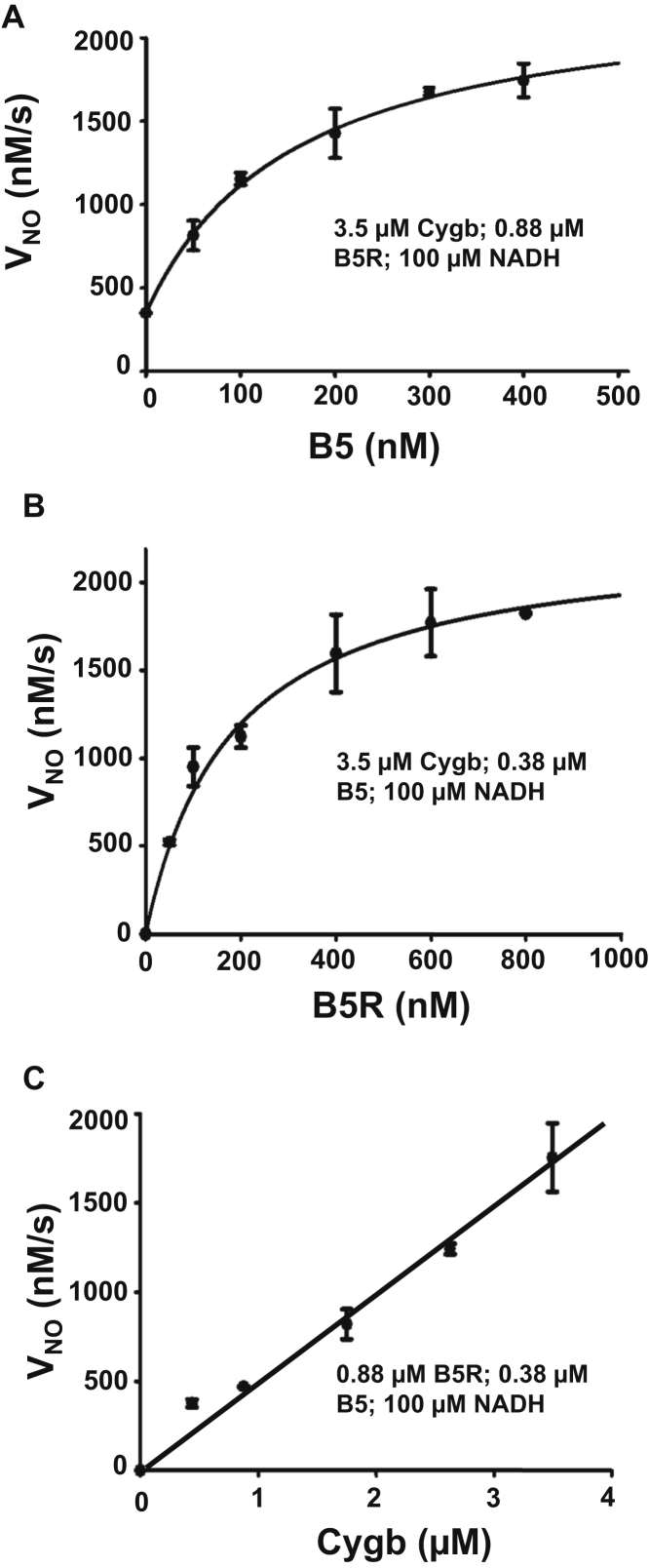
**Effect of varying B5, B5R, or Cygb levels on NO metabolism.** NO decay rate (*V*_NO_) was measured with variation of B5 (*A*), B5R (*B*), or Cygb (*C*) concentrations from the levels determined in SMCs. Solid lines in *A*–*B* show fit of the experimental data to the Michaelis–Menten equation: for (*A*) with K_m_’ = 149.5 nM for B5 and V_max_ = 2021 nM•s^−1^ with offset of 350 nM•s^−1^ observed at 0 [B5] due to the low rate of reduction of Cygb by B5R; and for (*B*) with K_m_’ = 130.5 nM for B5R and V_max_ = 2013 nMs^−1^ (*B*). Precise fitting was obtained with r^2^ of 0.997 and 0.950 respectively for *A*–*B*. Line in (*C*) is a linear fit with a slope of 510 nM•s^−1^/μM with r^2^ of 0.981. Experiments were performed and analyzed similar to Figure 8. Values shown are means ± SD with n = 3.

In order to determine the importance of the B5/B5R ratio on V_NO_, further experiments were performed assessing the effects of decreasing the B5 with fixed B5R or decreasing the B5R with fixed B5. These experiments model the effects of selectively decreasing the levels of either B5 or B5R as was achieved in the cellular knockdown experiments. We measured V_NO_ with cellular Cygb concentrations (3.5 μM) and B5R concentrations (0.88 μM) and varied the B5 concentration. As shown in Figure 10*A*, with fixed B5R, V_NO_ increased as a function of B5 concentration and showed partial saturation at high B5 concentration. Hyperbolic dependence was seen that could be fit by the Michaelis–Menten equation with K_m_’ = 149.5 ± 12.7 nM for B5 and V_max_=2021 ± 68 nM•s^−1^. This data predicts a maximum Cygb turnover number of 0.57 s^−1^. The small offset of 350 nM•s^−1^ observed in the absence of B5 is due to the low rate of direct reduction of Cygb by B5R. Variation of B5R concentration, with fixed B5 and Cygb (with cellular concentrations of 0.38 μM and 3.5 μM, respectively), also showed a hyperbolic dependence with saturation of V_NO_ at higher B5R concentrations (Fig. 10*B*). This data can also be fit using the Michaelis–Menten equation with K_m_’ = 130.5 ± 33.4 nM for B5R and V_max_ = 2013 ± 152 nM•s^−1^ with corresponding maximum turn over number of 0.58 s^−1^. This hyperbolic response of V_NO_ for B5 or B5R concentration change indicates that with increases or decreases from the SMC B5 or B5R concentrations with fixed Cygb, changes in V_NO_ would be less than linear. This suggests that with any approach to decrease cellular B5 or B5R, the decrease in observed V_NO_ would be less than the decrease in these protein levels. For example, a 90% decrease in the B5 concentration in SMCs is expected to decrease the V_NO_ by only 74% while a 90% decrease in B5R would only decrease the V_NO_ by 60% (Fig. 10, *A*–*B*).

We also carried out similar experiments varying the Cygb concentration measuring V_NO_ with B5 and B5R levels fixed at cellular levels. With decreasing Cygb concentration from the levels measured in SMCs of 3.5 μM to less than 1 μM, a linear decrease in V_NO_ was observed (Fig. 10*C*). The slope of this line has a value of 510 nM•s^−1^/μM Cygb. Therefore, the turnover number is calculated to be 0.51 s^−1^, which is consistent with the experiments summarized above and the value of 0.5 s^−1^ previously reported (16). Thus, with the levels of B5 and B5R present in SMCs, the rate of NOD is linearly dependent on the Cygb concentration.

## Discussion

Cygb was discovered about two decades ago as the fourth major globin expressed in mammals (14, 34, 35). While Cygb was first discovered in hepatic stellate cells, later it was found to be expressed in a variety of cells, including SMCs of vessels (19, 35, 36). Although this globin was originally found as a cancer marker (18, 37, 38), later it has been shown to have many other functions including enhancing progenitor cell proliferation (39), atherosclerosis (40), antioxidant protection (20) and NO metabolism (5, 8). Alterations in Cygb expression have been found to be associated with a variety of disease pathologies including cancer (41), atherosclerosis (42), hypertension (5) and fibrosis (43).

Following the discovery that it is highly expressed in the vascular smooth muscle, Cygb has received increased attention and proposed to have a key role in the regulation of vascular tone (4, 5, 7, 19). Recently, this has been confirmed from studies in mice where genetic knockout of Cygb was shown to result in lower vascular tone with lower blood pressure and decreased systemic vascular resistance due to enhanced NO-dependent vasodilaton (5). Vascular smooth muscle cells from various species, including humans, express Cygb (5, 19). Immunohistology studies have shown the expression of Cygb in the smooth muscle medial and adventitial layers of the aorta, while it is absent from the endothelium (5). From studies quantitating the levels of Cygb and other globins in smooth muscle cells, it has been confirmed that Cygb is highly expressed (5). Levels of Cygb were shown to be much higher than Mb in human aortic smooth muscle with values of ∼45 ng/10^6^ cells compared with ∼1 ng/10^6^ cells, respectively. Hb-α expression was trace or undetectable with levels >200-fold below those of Cygb. In addition, the rates of Cygb reduction and its resultant NO dioxygenation are >tenfold higher than those of Mb or Hb-α. In view of its high NOD activity and high expression levels in vascular SMCs, Cygb would be expected to have a major role in vascular NO metabolism and secondary regulation of vascular tone (5).

Whereas Cygb has many similarities to other globins such as Hb and Mb, including the classic three-over-three alpha helical globin fold, its biological and physiological functions are different from other globins. Among the globins, Cygb more efficiently degrades NO due to its higher rate of NOD (5, 7, 16, 29). Cygb has been shown to have an important role in NO metabolism in vascular SMCs and in regulation of NO flux across conduit and resistance vessels (5, 7, 28, 44, 45). Unlike Hb or Mb, Cygb is six-coordinate in both Fe^2+^ and Fe^3+^ states *via* axial imidazole nitrogen ligands from His81 and His113 in the absence of competing ligands. Therefore, any gasotransmitters such as O_2_ or NO have to displace a His ligand to bind to the heme Fe. In spite of this, O_2_ binds strongly to Cygb (46). In the pocket of the heme core, initial binding of O_2_ displaces the exchangeable histidine (His81) coordinated to the reduced Fe (Fe^2+^), and the heme dioxygen complex Fe^2+^-O_2_ is formed. This species is the precursor that dioxygenates the NO to NO_3_^−^ and is oxidized back to met-Cygb (Cygb-Fe^3+^), which is then reduced back to Fe^2+^, binding O_2_ to again form the precursor. This cycle continues as long as O_2_ and the required reducing equivalents are available. NOD requires active regeneration of oxy-Cygb-Fe^2+^. The process of Cygb-Fe^3+^ reduction to Cygb-Fe^2+^ has been extensively studied (7, 29). This reduction rate is on the order of 2 to 3 × 10^5^ M^−1^•s^−1^ for some enzymatic reducing systems, whereas for some chemical reductants it is on the order of 1 to 36 M^−1^•s^−1^ (29).

In the present work, we have performed a series of experiments in cultured vascular SMCs to determine the role of Cygb in the process of smooth muscle NO metabolism, as well as its regulation by the available O_2_ concentrations. Detailed evaluation of the reducing systems present in these cells and their relative role in the process of NOD was performed. As O_2_ is required for NOD with O_2_ levels critical for regulating NO metabolism by Cygb, we simultaneously monitored the NO and O_2_ concentrations. From studies of the isolated protein, it has been proposed that Cygb can function as an O_2_ sensor to provide feedback regulation leading to vasodilation under hypoxic conditions. The NOD rate of Cygb was shown to be O_2_-dependent, with a sharp decrease below 50 μM O_2_ and an apparent K_m_ of about 40 μM (7, 28). This sensitive O_2_ dependence was unique for Cygb and not seen for other globins such as Mb (28). In the current work measuring NO decay from SMCs, it was observed that NO consumption shows a hyperbolic dependence on the [O_2_], similar to the prior work with isolated Cygb in the presence of Asc or other reducing systems. However, the apparent K_m_ value for O_2_ in SMCs was observed to be 120 μM (Fig. 2*C*), which is higher than the value seen with purified Cygb and ascorbate as reductant (7, 28). As predicted by the related equation for the K_m_’ for O_2_, this apparent K_m_ is predicted to vary with the concentration of reducing system present as well as other factors (7). Since in SMCs, we see that B5/B5R is the main reducing system present, the observed apparent K_m_ value in SMCs would not necessarily be expected to be the same as in the prior study with the purified enzyme in the presence of ascorbate. This experimental data suggests that with a decrease of pO_2_ from arterial values, typically about 120 to 60 torr, the NOD rate as measured would decrease by nearly twofold. As the severity of hypoxia would further increase, the NOD rate would also greatly decrease. Thus, with cellular hypoxia in SMCs, as O_2_ levels decline, the NO dioxygenase rate would also decline, decreasing the rate of NO metabolism, which would raise NO levels that in vessels could serve to trigger compensatory vasodilation through increased activation of sGC. These studies support the concept that Cygb in smooth muscle can serve as an O_2_-dependent sensor that regulates vascular tone (7).

The reaction between reduced Cygb and O_2_ is much faster than the simple NO decomposition in the presence of [O_2_]. While both processes are second order in [NO], the rate constant for Cygb-mediated NOD is 2.2 × 10^7^ M^−1^ s^−1^ (7), while the apparent second-order rate constant for the reaction of NO with O_2_ is 2 × 10^3^ M^−1^ s^−1^ (47). Therefore, even at low O_2_ in the cell suspension (and therefore intracellular O_2_), still the major pathway of NO degradation will be from Cygb-mediated NOD to form NO_3_^−^. We have previously confirmed and have measured the major NO degradation product to be NO_3_^−^, formed by the NOD reaction (5). Furthermore, as we have published previously, in near anoxic conditions, Cygb can function as a nitrite reductase consuming nitrite to paradoxically generate NO (4). In this way Cygb serves as an O_2_ -dependent NO scavenger when cells are well oxygenated but produces NO when there is ischemia with severe hypoxia.

In the current studies from quantitative immunoblotting, we estimate that the Cygb concentration in the mouse aortic SMCs studies is ∼3.5 μM. This value is in the range of 2 to 4 μM as reported in prior studies (5). In these control SMCs in air with 3.5 × 10^6^ cells•ml^−1^, the NOD rate was measured to be 12.7 ± 0.3 nM•s^−1^. From experiments with siRNA-mediated knockdown of Cygb expression, it was demonstrated that 78% of measured NO metabolism in the murine aortic SMCs studied is Cygb-dependent (Table 1). This is consistent with prior studies in rat aortic or mesenteric artery SMCs, demonstrating that Cygb accounted for >75% of NO metabolism (5). Thus, our current studies confirm the important role of Cygb as the major NO dioxygenase in vascular SMCs.

**Table 1:** Role of Cygb, B5R, B5, and CPR in NOD activity in SMCs

Protein	Cellular concentration (μM)	KD (%)	Decrease in V_NO_ (%)	Correction factor for % KD and nonlinearity	Total NOD activity (%)
Cygb	3.5	87	68	1.15	78
B5R	0.88	95	53	1.28	68
B5	0.38	90	49	1.33	65
CPR	0.15	95	18	1.05	19

Questions and controversy remain regarding the specific process of Cygb reduction in SMCs and the relative importance of proposed chemical or enzymatic reducing systems. Various reducing equivalents and reducing systems have been identified in cells capable of reducing the oxidized form of Cygb-Fe^3+^ (metCygb) to Cygb- Fe^2+^ (8, 29). Spectrophotometric studies on the isolated protein have demonstrated that ascorbate can function as an effective reducing agent for Cygb supporting rapid NO consumption (7, 16, 28, 30). Interestingly, Cygb exhibits a unique reduction pattern with ascorbate. Whereas other globins, such as Mb, show irreversible reduction with ascorbate, Cygb shows a reversible reduction with reduced Cygb oxidized back to Fe^3+^ by rising levels of oxidized ascorbate, slowing the net rate of Cygb reduction (7, 28). This unique observation suggests that complex formation between Cygb and ascorbate may occur, as first hypothesized by Gardner (16), although to date there is a lack of direct experimental proof of this complex formation. The paradoxical reduction in V_NO_ seen in ascorbate-supplemented SMCs could also be due to ascorbate serving as an electron donor to the mitochondrial respiratory chain (complex IV) or to other redox reactions with possible indirect effects on NO consumption. Various flavin-containing reductase enzymes such as CPR and B5R have been shown to be highly effective in reduction of Cygb (29). As is the case for other globins, the NADH/cytochrome b5/cytochrome b5 reductase (B5/B5R) reduction pathway has been proposed to be of particular importance. The B5/B5R reducing system provides high reduction rates, while the rate for ascorbate is much lower; however, the concentration of ascorbate can reach millimolar levels in the cell (48), while expression levels of B5R or B5 would be expected to be orders of magnitude lower (5). Thus, major questions remained regarding the process of Cygb reduction in SMCs and the relative importance of the proposed reducing systems.

While from prior isolated protein studies, ascorbate at high concentrations has been shown to support the reduction of Cygb and its NOD activity, its role in the NOD function in SMCs is unclear and remains controversial (7, 16, 31). As noted above, whereas the rate of NOD by Cygb with ascorbate is about 10,000-fold lower than that seen with the enzyme reductases, its concentration can be much higher. Prior studies have measured the concentration of ascorbate in cultured cells without supplementation to be 40 to 50 μM (32). In view of the high reported K_m_ for Cygb reduction by ascorbate of 4.1 ± 0.5 mM and a Vmax′ of 1.48 ± 0.05 μM•s^−1^ (7, 16), in the absence of supplementation, the intrinsic ascorbate levels would not be expected to support much of the NOD function of Cygb. Consistent with this, in our experiments with ascorbate-depleted cells, grown in totally ascorbate-free media, there was no significant difference in the rate of NO decay compared with the control SMCs. Furthermore, supplementation with 5 mM ascorbate in the media during culture and also after harvesting the cells for measurements, a paradoxical 26% decrease was observed in the NO decay rate, rather than an increase. As discussed above, in view of the proposed binding site for ascorbate to Cygb in the proximity of the heme (16), as well as its much lower NOD rate compared with cellular reductases such as B5/B5R and CPR, the observed inhibition could be due to ascorbate binding partially impeding the interaction of the enzyme reductases with Cygb. Thus, ascorbate or a secondary oxidized ascorbate metabolite may function to inhibit the process of cellular NOD.

The role of the cellular reducing systems B5/B5R and CPR, previously shown to reduce Cygb and support NOD function in isolated protein studies, was evaluated by measuring their cellular levels and the effects of siRNA-mediated knockdown. From this quantitative immunoblotting, the cellular concentrations of these proteins were estimated to be: 0.88 μM for B5R; 0.38 μM for B5; and 0.15 μM CPR (Table 1). These levels are about four- to tenfold higher than those previously assumed for B5R and B5 (5, 28) and CPR (7). Also, interestingly, the level of B5R measured is higher than B5. In contrast to most previously published studies with isolated protein-based assays where B5/B5R ratios are maintained at least 10:1 to maximize the electron flux for a given amount of B5R (5, 29, 31), we observe a fractional value of ∼0.43 in SMCs. Thus, interestingly, B5 levels appear to be more limiting than B5R, and the relatively low B5/B5R ratio in SMCs, as well as the critical role of B5 in the efficient coupling of electron transport from B5R to Cygb, would render B5 levels of importance in regulating Cygb reduction.

As noted above, we observed that the levels of B5R are ∼ sixfold higher than CPR and the levels of B5 ∼ 2.5-fold higher than CPR. In addition, the reduction rates of Cygb by CPR and B5/B5R have been reported to be in a comparable range, 3.2 ± 0.1 × 10^5^ M^−1^ s^−1^ for CPR/NADPH (7) and 2.9 ± 1.4 × 10^5^ M^−1^ s^−1^ for B5/B5R/NADH (29). Therefore, based on the higher levels of B5R and B5 in the SMCs, B5/B5R would be expected to be the major intracellular reducing system supporting Cygb-mediated NOD function in SMCs. Indeed based on the measurements in SMCs with knockdown of CPR or B5R, only 19% of NO degradation was seen to be due to CPR, compared with 68% with B5R (Table 1).

In experiments with purified Cygb, B5R, and B5 at concentrations similar to those measured in the SMCs and in the presence of 100 μM NADH, *V*_NO_ was found to be 1741 ± 63 nM•s^−1^. This high *V*_NO_ was 140-fold higher than that determined in the SMC suspensions (Fig. 1). Since the packed volume of cells from suspensions with 3.5 × 10^6^ cells/ml was ∼0.7%, the predicted value for *V*_NO_ in the cell suspension would be ∼12.2 ± 0.4 nM•s^−1^, consistent with the value of 12.7 ± 0.3 nM•s^−1^ that was observed in the control SMC suspensions (Figs. 2 and 4). Thus, the cellular NOD rate was comparable with that with the purified proteins with similar *V*_NO_ at similar solution concentrations.

From the observed *V*_NO_ rates and the measured Cygb concentration in the SMCs, we can estimate the Cygb turnover number in SMCs. Considering the rate 12.7 ± 0.3 nM•s^−1^ that was observed in the control SMC suspensions with estimated mean Cygb concentration in these suspensions of 24.5 nM, we calculate a turnover number of 0.51 s^−1^, and this is consistent with isolated protein studies modeling the concentrations of Cygb, B5, and B5R measured in these cells as well as prior observations (16). Interestingly, from the experiments performed modeling the effects of changes in the cellular concentrations of Cygb, B5, and B5R and the observed V_max_, we saw that a maximum Cygb turnover number of 0.57 or 0.58 s^−1^ was obtained (Fig. 10). This suggests that the levels of B5 and B5R present in the SMCs are sufficient to provide ∼90% of maximum Cygb turnover rate in support of the process of NOD.

We observed that with knockdown of either B5R or B5, the rate of NO decay was greatly decreased compared with that in untreated control or scr-RNA treated cells. In addition, in CPR knockdown cells, a lesser effect was seen. With siRNA-mediated knockdown achieving ∼90% depletion of basal B5R or B5 expression, we observed that V_NO_ decreased by 53% and 49%, respectively. If the decrease in V_NO_ linearly follows the B5R and B5 concentrations, 58.9% and 54.4% decreases would be expected with 100% depletion, respectively. However, from isolated protein studies, the V_NO_ rates measured at cellular concentrations *versus* the rate with 90% depletion would decrease by only 85% and 71% for B5R and B5, respectively (Fig. 10, *A*–*B*).

From modeling the cellular protein concentrations, we observed that decreasing the concentration of Cygb, B5R, and B5, while maintaining their relative ratios with NADH fixed at 100 μM, the *V*_NO_ exponentially declined (Fig. 8*A*). This indicated that the higher concentrations of B5/B5R measured in cells are required to maximize the rate of Cygb reduction and the resulting *V*_NO_ (Fig. 8, *A*–*B*). Further experiments with fixed Cygb concentration and decreased levels of B5R and B5 while maintaining their relative ratios (Fig. 9) confirmed that the high levels of B5R and B5 observed are required to maximize V_NO_. Even at these high levels, only partial plateauing of V_NO_ was seen (Fig. 9*B*). With changing the levels of B5 or B5R alone, it was seen that at the highest concentrations, approaching those present in cells, partial saturation occurs with hyperbolic dependence (Fig. 10, *A*–*B*). This saturation seen for high levels of B5R is similar to that reported by Amdahl *et al.* (29) for Cygb reduction by B5/B5R. Interestingly, these experiments show that for the levels of Cygb and B5R present, there is no need for the B5/B5R ratio to be much higher as the observed V_NO_ rate is ∼90% of the maximum rate with ∼90% of the maximum Cygb turnover number achieved. Indeed, raising the ratio will not proportionately increase the NOD rate, as saturation is attained in B5 *versus* V_NO_ for given B5R and Cygb (Fig. 10*A*). In contrast, with decrease in Cygb concentration from cellular levels, a linear decrease in V_NO_ was seen (Fig. 10*C*).

**Figure 11:**
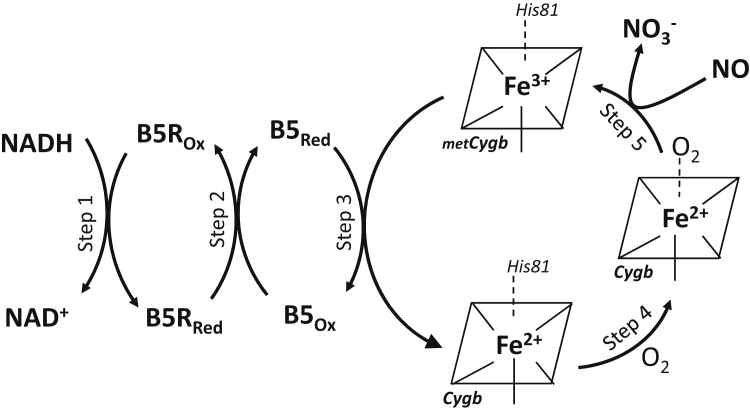
**The reaction steps of Cygb-mediated NO dioxygenation in vascular SMCs.** The reducing substrate NADH provides electrons to cytochrome b5 reductase (B5R) in Step 1 which, in turn, reduces cytochrome b5 (B5) in Step 2. The reduced B5 then reduces metCygb (Fe^3+^) to Cygb Fe^2+^ in Step 3, which binds O_2_ forming the oxyferrous species, CygbFe^2+^-O_2_ in Step 4, with displacement of the coordinated histidine (His). The oxyferrous Cygb species reacts with NO to form NO_3_^−^ in Step 5 oxidizing Cygb back to metCygb(Fe^3+^) species, which can cycle again.

The observed saturation in V_NO_ with variation of B5 and B5R as seen in Figure 10, *A*–*B*, shows that the NOD activity of Cygb in the presence of B5/B5R does not follow simple second-order kinetics. This NOD process includes reduction of Cygb-Fe^3+^ to Cygb-Fe^2+^, followed by the binding of O_2_ to form Cygb-Fe^2+^-O_2_, mediating NOD activity, in a multistep process. The five major sequential steps in the overall reaction are illustrated in Figure 11. As shown, a two-step coupled reductase system involving B5R reduction by NADH (Step 1) leads to B5 reduction by B5R (Step 2), followed by Cygb reduction by B5 (Step 3) that enables formation of the Cygb-Fe^2+^-O_2_ complex (Step 4) that, in turn, mediates NO conversion to NO_3_^−^ (Step 5). Of note, as previously reported, step 3 is the rate-limiting step in this series of reactions (7, 8, 29). We observe that the dependence of V_NO_ on B5 concentration can be precisely fit with an apparent Michaelis–Menten equation with a K_m_’ of 149.5 ± 12.7 nM and a V_max_ = 2021± 68 nM•s^−1^. Similarly, with varying B5R concentration, a K_m_’ of 130.5 ± 33.4 nM with V_max_ = 2013 ± 152 nM•s^−1^ was observed.

In addition to the B5/B5R reducing system, CPR was also present in SMCs, but levels were ∼sixfold lower than B5R and only ∼19% of NO metabolism was CPR-dependent. While NADH serves as the primary reducing substrate for B5R, NAPDH is the substrate for CPR (5, 7). From the sum of the B5/B5R and CPR pathways, we can estimate that all of the NO decay can be accounted for by these reductase pathways. Overall, these results indicate that the B5/B5R reducing system is the major redox system supporting the NOD activity of Cygb in SMCs, while there is also a small contribution from CPR. Interestingly, while 78% of the total NOD activity in SMCs could be attributed to Cygb, 68% was estimated to be due to B5R and 19% from CPR. Thus, our data suggests that these two reductase pathways are required for both Cygb-mediated and other processes of NO degradation. Other pathways of NOD include the globins myoglobin (Mb) and hemoglobin (Hb). We have previously reported that Mb levels in SMCs are ∼40-fold lower than Cyb, while Hb is >200-fold lower (5). Therefore, these other globins could have only a small effect on the process of NOD in SMCs. Recent studies have shown that B5R is the reductase that reduces sGC to the ferrous state enabling the binding of NO (49). Thus, sGC also requires B5R and may contribute to the process of NO decay in SMCs. Therefore, B5R would also be required to support these Cygb-independent NO metabolism pathways.

Overall, we see that the process of Cygb-mediated NOD in SMCs is rapid with a value of ∼1740 nM•s^−1^ observed for 1 μM NO. Since V_NO_ varies linearly with the NO concentration (7), rates of 174 nM•s^−1^ and 17.4 nM•s^−1^ would be predicted for lower physiological NO levels of 100 nM and 10 nM NO. In general, NO levels would be predicted to decay by tenfold in ∼1 s. This rapid rate of NO decay would be important for rapid regulation of physiological vascular tone and blood pressure as required for normal homeostasis, as well as alterations required for exercise or stress response. This rapid NOD process will enable rapid modulation of the levels of NO available to bind to sGC, allowing for rapid alterations in vessel tone, and also serve to decrease the rate and levels of peroxynitrite formed from the reaction of NO with superoxide, which proceeds at near diffusion rates (10, 11, 12). Thus, the Cygb-mediated process of NO decay would serve to prevent reactive nitrogen species formation and subsequent oxidative injury and nitration.

In conclusion, the present work establishes that the NO dioxygenase function of Cygb is responsible for the majority of NO metabolism in SMCs, and this is primarily regulated by the reduction of ferric Cygb to ferrous Cygb by B5/B5R, with a lesser contribution from CPR. Cellular ascorbate levels had no significant role in this process. The levels of Cygb, B5R, and B5 in vascular SMCs were measured and in the presence of excess NADH result in a high rate of NO decay. This rapid rate of NO decay linearly decreased with reduction in cellular Cygb levels, while the high levels of B5R and B5 present resulted in partial saturation. Cygb-mediated NO consumption in SMCs is O_2_-dependent, so that as O_2_ levels decline, the NOD rate also declines, serving to raise NO levels and trigger compensatory vasodilation. Therefore, Cygb, in the presence of its requisite B5/B5R reducing system, provides O_2_-dependent regulation of NO metabolism in vascular SMCs that, in turn, plays an important role in the modulation of vasodilation and vessel tone.

## Experimental procedures

### Reagents

All chemicals and reagents were of analytical grade and were purchased from Millipore Sigma (St Louis, MO), unless otherwise stated. A truncated and water-soluble form of human cytochrome p450 reductase (CPR) was a kind gift from Dr Lucy Waskell (University of Michigan V.A. Research Center, Ann Arbor) and stored in 20% glycerol solution.

### Preparation of NO stock solutions

NO solution was prepared as described previously (5, 28, 47). Briefly, in a fume hood, NO gas was scrubbed of NO_×_ impurities by passing through a U-shaped tube containing NaOH pellets and then through 1 M deaerated (bubbled with 100% argon) NaOH solution in a custom-designed apparatus using only glass or stainless steel tubing and fittings. The purified gas was collected by saturating a deaerated phosphate buffer solution (0.2 M potassium phosphate, pH 7.4) contained in a glass sampling flask (Kimble/Kontes, Vineland, NI) fitted with a septum. A gas-tight Hamilton syringe (Hamilton Robotics, Reno, NV) was used for anaerobic extraction of NO solution.

### Cell culture

Murine aortic SMCs were purchased from American Type Culture Collection (ATCC; CRL-2797) and maintained in RPMI 1640 medium (Invitrogen) supplemented with 10% fetal bovine serum (FBS; Invitrogen), penicillin (100 U/ml), and streptomycin (100 μg/ml) at 37 °C with a 5% CO_2_ atmosphere in a humidified incubator.

### Measurements of NO consumption by SMC suspensions

The rate of NO metabolism by normal SMCs of different cell numbers was assessed as previously described (5). Briefly, a Clark-type NO electrode (ISO-NOP, WPI, Sarasota, FL) was placed through one port on the side wall of a four-port water-jacketed electrochemical chamber (NOCHM-4, WPI, Sarasota, FL), while an oxygen electrode (ISO-OXY-2, WPI, Sarasota, FL) was placed through a second port on the side wall of the chamber for oxygen monitoring. Measurements were done at 37 °C in air-equilibrated buffer continuously stirred with a magnetic bar. The rate of NO decay in the buffer solution was measured before adding the SMCs into the solution following injection of NO to achieve an initial concentration of 1.0 μM using a 10 μl gas-tight Hamilton syringe. The oxygen level was maintained at ∼240 μM (as simultaneously monitored by the O_2_ electrode) *via* passage of purified air in the head space of the chamber. For depleting O_2_ in experimental cell suspensions, pure argon gas was passed over the solution, as previously described (7). To correct for the low basal rate of NO decay due to the reaction of NO and O_2_ in the buffer, a correction curve was constructed from measurements of this kinetics in isolated buffer solutions for the given amount of NO in the presence of varying O_2_ concentrations and these measured background V_NO_ values were subtracted from the observed values measured in the cell suspensions.

The rate of NO metabolism by control isolated cultured SMCs or scrambled siRNA (scr-RNA) treated, Cygb knockdown (Cygb siRNA), B5R knockdown (B5R siRNA), B5 knockdown (B5 siRNA), and CPR knockdown (CPR siRNA) SMCs was assessed as described above.

### Knockdown of Cygb, B5R, B5, or CPR in SMCs

SMCs were transfected with Cygb siRNA (Santa Cruz; sc-45548), B5R siRNA (Santa Cruz; sc-62174), B5 siRNA (Santa Cruz; sc-37378), or CPR siRNA (Santa Cruz; sc-35148) using Lipofectamine RNAiMAX (Invitrogen) according to the manufacturer's recommendations. Briefly, Lipofectamine RNAiMAX was mixed gently with antibiotic-free Opti-MEM medium (Invitrogen) in a ratio of 6 μl:100 μl for each 1 ml growth medium followed by incubation at room temperature for 5 min. An aliquot of Cygb, B5R, B5, or CPR siRNA was mixed with the Lipofectamine RNAiMAX/Opti-MEM mixture to reach a final concentration of 100 nM. Following incubation at room temperature for 30 min, 1 ml of each mixture was added to a separate 150 × 25 mm culture plate containing SMCs exponentially growing in 9 ml of antibiotic-free Opti-MEM medium. Cygb, B5R, B5, or CPR siRNA and the corresponding scrambled siRNA (scr-RNA)-transfected cells were incubated at 37 °C in a 5% CO_2_-humidified incubator. Seven hours later, Opti-MEM medium was changed to RPMI 1640 complete medium to provide essential nutrients and growth factors for optimal growth and cell survival. Forty-eight hours post transfection, the cells were collected for further studies, and protein expression was evaluated by western blotting.

### Western blotting

Whole-cell lysates or pure proteins, in RIPA lysis buffer, were quantitated using a Bio-Rad DC protein assay kit. Proteins were separated on SDS-polyacrylamide gel (4–20%) at 125 V and then transferred onto a PVDF membrane using an Xcell II Blot Module (Invitrogen) at a constant 25 V for 90 min. The following antibodies were used: rabbit polyclonal anti-Cygb, with similar reactivity to mouse, rat and human Cygb (Invitrogen; PA5-75671, diluted 1:200); mouse monoclonal anti-CyB5R3 raised against amino acids 1 to 60 at the N-terminus of human CyB5R3 with sequence homology and similar reactivity in mouse, rat, and human (Santa Cruz; sc-398043, diluted 1:200); rabbit polyclonal anti-CyB5 to amino acids 21 to 134 at the C-terminus of human CyB5 (Santa Cruz; sc-33174, diluted 1:200); mouse monoclonal anti-CPR raised against amino acids 1 to 300 of human CPR with sequence homology and similar reactivity in mouse, rat, and human (Santa Cruz; sc-25270, diluted 1:100) and mouse monoclonal anti-actin (Santa Cruz; sc-47778; diluted 1:400). Membranes were blocked with 5% dried milk in Tris-buffered saline containing 0.05% Tween 20 (TBST) for 1 h at room temperature and incubated overnight with primary antibodies at 4 °C. Membranes were then washed three times in TBST, incubated for 1 h with horseradish peroxidase-conjugated secondary antibody in TBST at room temperature, and again washed three times in TBST. Protein bands were then detected with ECL Western Blotting detection reagents (Amersham Biosciences) and exposed to an X-ray film. Protein band densities were quantified by a high-resolution Pharos FX Plus Molecular Imager (Bio-Rad). The protein expression levels were obtained by quantization of band intensities of each protein compared with band intensities of the corresponding pure protein standards run in parallel.

### Expression and purification of Cygb

The expression plasmid for Cygb (human Cygb cDNA in pET17b) was obtained from Dr Paul Gardner (Miami Valley Biotech, Dayton, OH) as a kind gift and transformed into the *E. coli* strain C41(DE3)pLysS according to the supplier's protocol (Lucigen Corporation, WI). Cygb was purified according to published literature (5, 16) with some modifications to maximize the yield. Briefly, cells were grown overnight in 10 ml LB medium containing 10 mM glucose, 100 μg/ml ampicillin, and 34 μg/ml chloramphenicol in an incubator shaker with 180 rpm speed at 37 °C. The grown culture was washed with fresh media, transferred into a 5-L flask containing 1 L Terrific Broth (TB) (47.6 g/l) supplemented with glycerol (8 ml/l), catalase (5 U/ml), ampicillin (100 μg/ml), and chloramphenicol (34 μg/ml), and allowed to grow in the shaker (set to 180 rpm) at 37 °C. Once the OD at 550 nm becomes ∼1.1, δ-aminolevulinic acid (δ-ALA) (0.5 mM) was added and then the culture was induced by isopropyl-1-thio-D-galactopyranoside (IPTG) (0.5 mM). Cells were allowed to continue growing overnight at 27 °C with minimal aeration and a shaking speed of 140 rpm. Cells were harvested by centrifugation (4000 rpm, at 4 °C for 30 min) and washed with 50 mM potassium phosphate buffer (pH 7.8) containing 0.1 mM EDTA. Cell pellets were frozen and thawed twice, suspended in lysis buffer (20 mM potassium phosphate buffer), pH 7, 1 mM ETDA, one Roche protease inhibitor tablet, deoxyribonuclease-I (0.05 mg/ml), and a pinch of lysozyme, and lysed by sonication (five cycles, each 1 min at 60 Hz, at 4 °C with 10 min interval) using a Branson Digital Sonifier. The crude cell extract was removed by centrifugation (45,000, at 4 °C) for 1 h, and the supernatant was dialyzed twice against 2 L of 1 mM potassium phosphate buffer (pH 7) at 4 °C. After heating at 65 °C for 10 min, the dialyzed cell extract was centrifuged at 45,000*g* for 1 h at 4 °C, and the protein was subsequently purified with an Äkta Purifier FPLC system (GE Healthcare) using a series of columns including a HiPrep 16/10 Q-Sepharose FF, HiPrep 26/60 Sephacryl S-300 High Resolution, and Superdex S-75 10/30 GL columns using the UNICORN software. Cygb purity was checked and the protein was concentrated using Amicon Ultra Centrifugal filters (Millipore) with a 10 kDa molecular weight cutoff. The protein concentration was determined by Bradford method (50) using bovine serum albumin as a standard. Finally, the protein was stored in 30 μl aliquots at −80 °C.

### Expression and purification of cytochrome b5 reductase (B5R)

The expression plasmid for b5 reductase (human B5R cDNA in pET100), with an N-terminal hexahistidine (His_6_) tag, was obtained from Dr Lauren Trepanier (University of Wisconsin, Madison). The plasmid was transformed into the *E. coli* strain BL21(DE3) as described previously and plated on LB agar containing 100 μg/ml ampicillin. A single colony from the agar plate was used to inoculate 20 ml of TB (47.6 g/l) containing 100 μg/ml ampicillin and grown at 37 °C for 4 h in a shaker incubator set at 180 rpm speed. About 5 ml of culture was then transferred into a 5 l flask containing 1 l TB (47.6 g/l) supplemented with riboflavin (0.1 mM) and 100 μg/ml ampicillin and continuing growth in a shaker with 180 rpm speed at 37 °C until the OD at 600 nm becomes ∼0.6. The culture was induced by IPTG (0.5 mM), and the expression was continued overnight at 27 °C with a reduced shaking speed of 130 rpm. Cells were then harvested by centrifugation at 4000 rpm for 45 min at 4 °C. Cell pellets were washed with fresh TB medium containing 100 μg/ml ampicillin and exposed to two freeze–thaw cycles, resuspended in ∼200 ml lysis buffer (40 mM HEPES buffer, pH 7.4, 10 mM imidazole, 300 mM NaCl, 1 mM DTT), and incubated with MgCl_2_ (1 mM), lysozyme (1 mg/ml), and one Roche protease inhibitor tablet for about 10 min prior to disruption by sonication. Soluble protein solution was separated and purified with an Äkta Purifier FPLC system (GE Healthcare) by loading on to a nickel nitrilotriacetic acid (Ni-NTA) column using a sample pump, washed with Buffer A (40 mM HEPES buffer, pH 7.4, 10 mM imidazole, 300 mM NaCl, 1 mM DTT). Bound protein was eluted with Buffer B (40 mM HEPES buffer, pH 7.4, 250 mM imidazole, 300 mM NaCl, 1 mM DTT) and then concentrated by centrifugation (3000 rpm, at 4 °C) using Amicon Ultra 4 filters (Millipore) with a 10 kDa MW cutoff. Using the same FPLC system, the protein was further purified by loading on to a Superdex 75 10/30 GL column pre-equilibrated with Buffer C (50 mM potassium phosphate buffer, pH 7.8, containing 0.1 mM EDTA) and eluted with same buffer. Fractions containing the B5R were combined and concentrated as described previously. The concentration of the protein was determined and stored in 30 μl aliquots at −80 °C.

### Expression and purification of cytochrome b5 (B5)

Recombinant soluble cytochrome b_5_ was purified from an expression plasmid kindly provided by Dr Lucy Waskell (Department of Anesthesiology, Michigan Medicine, Ann Arbor, Michigan) and transformed into *E. coli* strain C43(DE3)pLysS. Cells were grown overnight in a 4 l flask in an incubator shaker at 37 °C in 1 l of Terrific Broth (47.6 g/l) supplemented with glycerin (8 ml/l), ampicillin (0.2 g/l), and chloramphenicol (0.05 g/l). The following morning, the flask of cells was placed in the cold room for 30 min, then 0.5 mM 5-aminolevulinic acid (a heme precursor) was added to the cells (0.083 g/l), the cells were placed back in the incubator shaker at 28 °C for 30 min, followed by induction with 100 μM IPTG (0.024 g/l). The bacteria were grown for an additional 5 h at 28 °C. The cells were harvested by centrifugation (3000 rpm for 30 min), and the cell pellet was resolubilized in 100 ml of 50 mM TRIS-HCl pH 7.5, 1 mM EDTA, a pinch of lysozyme and deoxyribonuclease I, and Roche Complete Protease Inhibitor tablets. The cells were placed in a stainless steel 250 ml beaker immersed in ice and lysed by sonication with a Branson Digital Sonifier equipped with a ½” horn, using four 2 min repetitions with 10 min cool-down steps between each repetition. Insoluble matter was removed by centrifugation at 45,000*g* for 1 h in a high-speed centrifuge. The supernatant was added to a 250 ml beaker on ice, and a hemin solution (0.025 g hemin dissolved in 0.5 ml of 0.1 N NaOH) was added dropwise with stirring over a 30 min time period. The solution was left in a cold box with gentle stirring overnight. The next morning, the solution was centrifuged at 45,000*g* for 1 h to remove precipitated hemin. Further purification was performed with a GE Healthcare AKTA Purifier system equipped with a sample pump (GE Healthcare, Piscataway, NJ, USA) for sample loading. A HiPrep 16/10 DEAE FF anion-exchange column (GE Healthcare) was run with sodium chloride gradient elution (0 mM NaCl to 500 mM NaCl). Protein fractions with an A_414nm_/A_280nm_ ratio >3.5 were pooled and concentrated to ∼500 μl. This concentrated protein was further purified with a Superdex 200 10/300 GL high-resolution size-exclusion column (GE Healthcare) eluted with 50 mM Tris/HCl, pH 7.5, and 0.1 mM EDTA. The protein was concentrated and stored in 50 μl aliquots at −80 °C for later use.

### Estimation of cellular protein concentrations

The expression levels of Cygb, B5R, B5, and CPR were determined from the measurements of the levels of protein expression by quantitative immunoblotting *versus* recombinant protein standards for each protein prepared as described above. The cellular concentrations were determined from the ratio of the individual protein to the total protein multiplied by the total cellular protein concentration. Total cellular protein concentrations were determined by the Bradford method (50) and cell volume estimated by morphometry of isolated viable cells.

### Measurements of NO consumption for isolated Cygb, B5R, and B5

The rate of NO decay from cellular levels of Cygb, B5R, and B5, or fractional dilutions of these levels, was determined as previously described (5, 7), in a four-port water-jacketed electrochemical chamber (NOCHM-4 from WPI, Sarasota, FL) containing 2 ml of phosphate-buffered saline (pH 7.0) at 37 °C. The solution was rapidly stirred with a magnetic stirrer throughout the experiment. A NO electrode and an O_2_ electrode (WPI) were placed in the chamber through two ports in the side wall. The two electrodes were connected to an Apollo 4000 electrochemical instrument (WPI). After electrode stabilization, NO was injected into the aerated buffer solution (under room air). When the NO concentration decreased to baseline, NADH (100 μM) and SOD (6 μM) were added to the chamber, followed by injections of NO into the solution to measure the control rate of NO consumption in room air. After returning to base line, Cygb (3.5 μM), B5R (0.8 μM), and B5 (0.4 μM) were added to the solution, followed by bolus injections of NO. Afterward, the reaction mixture was serially diluted with buffer starting with 1.5-fold dilution to ∼81-fold dilution, while NADH was maintained at 100 μM. A concentration of 1 μM NO was used in all the cell experiments, while assays with purified proteins required either 1 μM NO or 10 μM to observe a measureable NO peak. The V_NO_ measured was normalized to 1 μM to compare results. From the recorded NO concentration curve, the rate of NO consumption for each NO peak was determined. Experiments were also similarly performed with dilution of both B5/B5R with a fixed ratio defined by their SMC levels and Cygb fixed at 3.5 μM. Similarly, the effect of isolated decreases in B5R, B5, or Cygb concentrations from their levels measured in SMCs was also performed.

### Statistical analysis

All measurements were performed at least three times for each data point. The values in the text are provided as mean ± S.E.M. Statistical significance of difference was evaluated by the student's *t* test or ANOVA analysis of variance. All data analyses and model evaluations were performed using Sigmaplot 13, with customized transforms.

## Data availability

All the data are included in the article.

## Conflict of interest

The authors declare that they have no conflicts of interest with the contents of this article.
